# Cloning of novel bacterial xylanases from lignocellulose-enriched compost metagenomic libraries

**DOI:** 10.1186/s13568-019-0847-9

**Published:** 2019-08-05

**Authors:** Simo Ellilä, Paul Bromann, Mari Nyyssönen, Merja Itävaara, Anu Koivula, Lars Paulin, Kristiina Kruus

**Affiliations:** 1 VTT Technical Research Centre of Finland, P.O. Box 1000, 02044 Vuorimiehentie, Espoo, Finland; 2 Institute of Biotechnology, University of Helsinki, P.O.Box 56, 00014 Helsinki, Finland

**Keywords:** Xylanase, Metagenomics, Lignocellulose, Compost, Screening, Cloning

## Abstract

Xylanases are in important class of industrial enzymes that are essential for the complete hydrolysis of lignocellulosic biomass into fermentable sugars. In the present study, we report the cloning of novel xylanases with interesting properties from compost metagenomics libraries. Controlled composting of lignocellulosic materials was used to enrich the microbial population in lignocellulolytic organisms. DNA extracted from the compost samples was used to construct metagenomics libraries, which were screened for xylanase activity. In total, 40 clones exhibiting xylanase activity were identified and the thermostability of the discovered xylanases was assayed directly from the library clones. Five genes, including one belonging to the more rare family GH8, were selected for subcloning and the enzymes were expressed in recombinant form in *E. coli*. Preliminary characterization of the metagenome-derived xylanases revealed interesting properties of the novel enzymes, such as high thermostability and specific activity, and differences in hydrolysis profiles. One enzyme was found to perform better than a standard *Trichoderma reesei* xylanase in the hydrolysis of lignocellulose at elevated temperatures.

## Introduction

Lignocellulosic biomass represents an attractive raw material base for the production of alternative fuels in the future due to its great abundance and renewability (Geddes et al. 2011). Producing renewable fuels and chemicals from lignocellulosic biomass along the biochemical conversion route requires the hydrolysis of the polysaccharide components of biomass, cellulose and hemicellulose, into their constituent sugars. Enzymatic hydrolysis has been identified as the most efficient means to this end (Girio et al. 2010), but current enzyme systems remain inefficient and consequently high doses of expensive enzymes are required. Enzymatic hydrolysis is cited as one of the limiting factors in the production of lignocellulosic bioethanol (Horn et al. 2012; Klein-Marcuschamer et al. 2012), and the development of more efficient biocatalysts is therefore clearly a priority for future commercial production. In particular, enzymes with good thermal stability considered crucial for applications (Viikari et al. 2007; Kumar et al. 2018, 2019).

Xylanases (endo-β-1,4-xylanases, EC 3.2.1.8) are an important class of lignocellulose degrading enzymes that cleave the β-1,4-glycosidic backbone linkages in xylan, the most prominent non-cellulosic polysaccharide in the cell walls of land plants (Girio et al. 2010). Xylan is especially abundant in the cell walls of hardwoods and grasses where it can represent up to 35% of total dry mass (Saha 2003). Xylanases are grouped into families of glycosyl hydrolases (GH) based on sequence homology (Henrissat 1991). The first xylanases to be discovered belonged to families GH10 and GH11, and these families remain clearly the best characterized. Families GH10 and GH11 both contain over 200 characterized members and have over 30 structure entries listed in PDB according to the carbohydrate active enzymes (CAZy) database (Cantarel et al. 2009). More recently, family GH30 has been found to include several xylanases (St John et al. 2010), many of which appear to be active specifically on glucuronoxylan. A handful of xylanases have also been reported from family GH8 (Pollet et al. 2010). Xylanases are often multidomain proteins, containing one or more additional domains such as carbohydrate-binding modules.

Xylanases are already used in several industrial applications ranging from the manufacture of food and feed to the bleaching of kraft pulp (Kumar et al. 2016, 2018). Xylanases are also important components of enzyme preparations currently on the market and utilized in commercial lignocellulosic bioethanol plants. The most common enzyme mixtures are derived from the fungus *Trichoderma reesei*, which can secrete large amounts of enzymes (Merino and Cherry 2007). However, as *T. reesei* is a mesophilic fungus, its enzymes can suffer from low stability when confronted with high temperatures and/or inhibitor concentrations (Rahikainen et al. 2013). There is therefore a clear need for more thermostable xylanases. Such enzymes can be isolated from thermophilic organisms and engineered for improved thermostability, as reviewed by Kumar et al. (2018).

While we still struggle to deconstruct lignocellulose in a controlled manner, a wide variety of fungi and bacteria are capable of using it as an energy and carbon source in nature (Rubin 2008). Metagenomics methodology allows the genetic study of these microbes directly from their habitats and grants access to the enormous biocatalytic diversity that they represent (Ferrer et al. 2009). Both activity- and sequence-based metagenomics methods have proven excellent in uncovering novel lignocellulose-degrading enzymes (Montella et al. 2016). In the present study, we report the cloning of novel bacterial xylanases from metagenomic libraries derived from the composting of lignocellulosic materials. Several interesting enzymes were uncovered, including a thermostable GH11 xylanase performing better than Xyn11A from *T. reesei* in the hydrolysis of hydrothermally pre-treated wheat straw at elevated temperatures.

## Materials and methods

### Enrichment of lignocellulosic microbes by controlled composting

Composting was conducted under controlled conditions (The CEN standard 14046) at 37 °C and 50 °C. Steam exploded spruce (from the University of Lund), cutter chips and Whitman filter paper were used in separate composts to enrich lignocellulolytic species in the microbial communities. Triplicate composts were run per substrate.

The biodegradation of the substrates was followed by quantitative online measurement of CO_2_ evolution for 20 weeks. One of three replicate composting bioreactors was sampled regularly and the samples were frozen at − 80 °C for DNA extraction. Total DNA was isolated using the PowerSoil DNA isolation kit (MoBio Laboratories) and the bacterial and fungal communities were monitored using 16S and 18S rRNA gene-targeting PCR-DGGE. The other two replicates ran untouched to obtain information about the biodegradability of substrates. Samples for constructing the metagenomic libraries were selected based on substrate biodegradation, and bacterial and fungal community succession. Samples from several time points and enrichment conditions were pooled for library construction.

### Construction of metagenomic DNA libraries

For the construction of large-insert metagenomic libraries, high molecular weight DNA was isolated by enzymatic lysis followed by hot phenol treatment and the isolated DNA was purified with pulse-field gel electrophoresis as described in Kielak et al. (2009). Metagenomic libraries were prepared from gel-extracted DNA with the CopyControl Fosmid Library Production kit (Epicentre) according to manufacturer’s instructions.

Two separate libraries, FOS-37 °C-CEL and FOS-50 °C-CEL, were prepared from DNA samples from 37 to 50 °C composts, respectively. Average insert size and library size was estimated from NotI restriction digests of fosmids isolated from 18 to 40 random clones. Before screening, the libraries were amplified to 10^9^ clones. The amplified libraries were stored in 20% glycerol at − 80 °C.

A separate plasmid library (PLA-50 °C-CEL) was prepared from samples from the 50 °C composts as follows: DNA was isolated using the PowerSoil kit (MoBio), blunt-ended using T4 polymerase, A-overhangs added using Taq polymerase, dephosphorylated using antarctic phosphatase (NEB) and finally TOPO-gated into the pCR2.1-TOPO vector (Invitrogen). The TOPO-gations were transformed into OmniMAX 2-T1 cells. The library size was estimated in a similar manner to the fosmid library, but using the restriction enzymes *Eco*RI/*Nco*I.

### Screening of metagenomic libraries for xylanases

The functional screen for xylanase activity was conducted on LB-agar plates containing 0.1% (w/v) Azurin-crosslinked xylan from oat spelt (AZCL-xylan, Megazyme). For the screening of the fosmid libraries, Copycontrol Fosmid Autoinduction Solution (Epicentre, USA) was added to screening plates in order to increase assay sensitivity. The screen was conducted at tenfold coverage of each primary library clone number to provide the maximal amount of unique xylanase-positive clones. *E. coli* clones surrounded by blue halos were picked, repeat plated and unique clones isolated using restriction analysis.

### Sequencing and sequence analysis

The BigDye^*®*^ Terminator v3.1 Cycle Sequencing Kit and an ABI Prism 3100 Genetic Analyzer (Applied Biosystems) were used to sequence all plasmids and fosmid terminal sequences. Fosmid clones identified in the xylanase-activity screen were pooled and sequenced using a 454 GS FLX Titanium instrument (Roche). The reads were assembled into contigs using Newbler (v. 2.0.00.20) and assigned to specific clones by aligning with the vector end sequences generated using Sanger sequencing. All assembled contigs above 1 kb are deposited in GenBank under accessions KX236210.1–KX236308.1.

Analysis and annotation of the available vector sequences was performed using Geneious Pro software (Kearse et al. 2012). A downloadable version of the carbohydrate active enzymes (CAZy) database was retrieved from the CAZymes Analysis Toolkit website (Park et al. 2010). The obtained vector sequences were used in standard BlastP queries against the whole CAZy database to locate carbohydrate active enzymes (CAZymes). Open reading frames (ORFs) were predicted using Glimmer 3 (Delcher et al. 2007). Putative xylanase-encoding ORFs were used in new BlastP queries against the CAZy database to find closest homologues, and in many cases, an annotation for associated domains. Signal sequence prediction was performed using SignalP Server (Petersen et al. 2011). Annotated xylanase sequences were deposited in GenBank under accessions MF171165.1–MF171184.1.

### Initial thermostability assay from cell lysates

Whole cell lysates for all unique xylanase-positive *E. coli* clones were prepared from 500 mL cultures using standard methods. In brief, when OD_600_ of the cultures reached 0.7, enzyme expression was induced using 1 mL of CopyControl Induction Solution (fosmid clones) or IPTG (plasmid clones). The cells were harvested 15–20 h (fosmid clones) or 3 h (plasmid clone) post-induction by centrifugation and resuspended in 5 mL volume of assay buffer (50 mM Na-citrate, pH 6.5) and lysed by sonication. Cellular debris was pelleted by centrifugation, and the supernatants (cell lysates) collected and stored at − 20 °C.

Xylanase activity from the whole cell lysates was measured using the fluorescence-based EnzCheck Ultra Xylanase Assay Kit (Invitrogen), according to the manufacturer’s instructions. The substrate used in the kit is 6,8-difluoro-4-methylumbelliferyl β-d-xylobioside (DiFMUX_2_). Reactions were carried out on microplates in 50 mM Na-citrate pH 5 at 40 °C for 30 min, with fluorescence measured at 40-s intervals. The thermostability of the xylanases in the cell lysates was assayed by incubating the lysates in water baths at given temperatures, taking samples at intervals, quantifying the xylanase activity using the EnzCheck assay and comparing to a pre-incubation reference to calculate residual activity.

### Subcloning

Fosmid (or plasmid) DNA was recovered from 5 mL cultures of each clone using the QIAprep Spin Miniprep kit (Qiagen) and used as templates for PCR. Primers were designed for the amplification of the selected genes (without signal sequences) and incorporation of the genes into the pASK-IBA16 expression vector (IBA GmbH). PCR-amplification of the xylanase genes was done using the iProof high-fidelity DNA polymerase (Bio-Rad). Cloning was performed using the recombination-based In-Fusion Advantage PCR cloning system (Clontech) and the completed reactions were used to transform Stellar competent *E. coli* cells (Clontech) according to manufacturer’s instructions. The constructed expression vectors were recovered using the QIAprep kit and correct sequences verified by Sanger sequencing.

### Expression and purification of recombinant xylanases

The subcloned plasmids were used to transform XL-1 Blue *E. coli* cells (Stratagene) using standard procedures. The clones were grown in LB medium containing 100 μg/mL carbenicillin in 1.5 L volume at 30 °C with 250 rpm shaking. At OD_600_ ≥ 0.7 the expression of the recombinant genes was induced by the addition of anhydrotetracycline (Clontech). After 2 h, the cells were harvested by centrifugation, washed with 90 mL of 50 mM Tris/HCl pH 8.0 and pelleted by centrifugation.

To recover the periplasmic protein, the cell pellets were resuspended in 50 mL of 50 mM Tris/HCl pH 8.0. To these suspensions sucrose, EDTA and lysozyme were added to final concentrations of 20% (w/v), 1 mM and 0.5 mg/mL, respectively. The suspensions were kept on slow rotation for 30 min at room temperature to allow cell wall lysis, followed by addition of 50 mL of ice-cold water. The suspension was kept on ice for 15 min with gentle intermittent inversions. The spheroblasts were pelleted by centrifugation and the supernatants (the periplasmic extracts) transferred to a fresh tube.

The periplasmic extracts were loaded onto 25 mL StrepTactin Sepharose High Performance columns (Invitrogen) and purified using an ÄKTAmicro purification system (GE Healthcare). Fractions were gathered and samples run on precast SDS-PAGE gels and visualized using the Criterion Stain Free Imager (Bio-Rad). Fractions containing visible bands of correct size were pooled and concentrated to a final volume of 3 mL using Vivaspin 20 (GE Healthcare) sample concentrators, with a cut-off of 10 kDa. The buffer was exchanged for 50 mM Na-citrate pH 5.0 using Econo-Pac 10DG desalting columns (Bio-Rad), and the concentrated purified recombinant xylanases stored at − 20 °C for further study. The protein concentrations of the purified proteins were measured using the Lowry-based Bio-Rad DC II protein assay kit using BSA as standard.

### Preliminary characterization of novel xylanases

Xylanase activity of the recombinant enzymes was measured on 1% birchwood xylan (Roth) in 50 mM Na-citrate pH 5.0. The standard reaction was conducted at 50 °C for 5 min, and released reducing sugars quantified using DNS, using xylose as standard. One unit of catalytic activity (U) was defined as the amount of enzyme capable of releasing 1 µmol of reducing sugar from xylan per minute under the assay conditions and standard errors calculated from triplicate reactions. This assay was used in further applications unless otherwise noted.

The pH-profiles of the xylanases were determined using three buffer systems: Na_2_HPO_4_/citric acid (McIlvane’s buffers—pH 3.0–6.5), Tris/HCl (pH 7.0–8.5), and glycine/NaOH (9.0–10.5). Buffers were prepared for 0.5 pH unit increments and 1% (w/v) birchwood xylan (Roth) solutions prepared in each buffer. Xylanase activity was then determined using the standard method.

To assay the optimal temperature for each xylanase, reactions were conducted at different temperatures over a range 20–90 °C, with buffer pH adjusted to be optimal for each enzyme based on the previous assay. Thermostability was assayed by incubating the enzymes in water baths at 60 °C and 70 °C and sampling at regular interval to quantify residual xylanase activity.

The activity of xylanases was quantified on three substrates: birchwood glucuronoxylan (Roth), wheat arabinoxylan (Megazyme) and carboxymethyl cellulose (CMC—Sigma). All substrates were prepared as 1% solutions in 50 mM Na-citrate buffers of optimal pH for each enzyme. Activity toward each substrate was measured as previously with glucose serving as standard for CMC activity.

To study the products of birchwood xylan hydrolysis, the standard xylanase assay was performed for 18 h instead of 5 min using 1 U/mL enzyme, and the released reaction products were analysed using high-performance liquid chromatography (HPLC). A CarboPac PA 1 column was used on an ICS-3000 ion chromatography system (Dionex). Xylo-oligosaccharides of a DP of 1–6 (xylose to xylohexaose—Megazyme) were used as standards.

### Hydrolysis of hydrothermally pre-treated wheat straw

To assess the enzymes’ efficiency on a technical lignocellulosic substrate, steam-exploded wheat straw, the xylanases were used as components of cellulolytic enzyme mixtures. For hydrolysis at 45 °C, an enzyme mixture comprising *T. reesei* enzymes was created by combining CBHI (TrCel7A), EGII (TrCel5A) and XYN2 (TrXyn11A) at a mass ratio of 70:20:10, and the enzyme mixture used in hydrolysis at an overall dose of 16 mg/g dry matter. β-Glucosidase (Cel3A) from *Aspergillus niger* was further dosed at an activity based concentration of 30 U/g dry matter. Similar enzyme mixtures were prepared with the purified metagenomic xylanases replacing the *T. reesei* xylanase (Xyn11A), while in one mixture xylanase was omitted. The purification of the reference enzymes has been described elsewhere (Suurnäkki et al. 2000).

Separate hydrolysis reactions were performed at a higher temperature (55 °C) using the two metagenomic xylanases that were found to be thermostable. The reference mixture of thermostable enzymes comprised CBHI from *Acremonium thermophilum* (AtCel7A), CBHII from *Clostridium thermocellum* (CtCel6A) and EGII from *Thermoascus aurantiacus* (TaCel5A) (Viikari et al. 2007), kindly provided by ROAL Oy. The enzymes were used at an overall dose of 10 mg/g dry matter at a ratio 50:20:20:10 (CBHI:CBHII:EGII:Xylanase) and β-glucosidase from *T*. *aurantiacus* (TaCel3A) added at 30 U/g dry matter.

The substrate used was wheat straw thermally pretreated at 190 °C for 15 min (BioGold—Estonia), prepared as a homogenous slurry in 50 mM Na-citrate pH 5.0 using an immersion blender. Triplicate hydrolysis reactions were performed in 500 µL volume in microcentrifuge tubes. Reactions were terminated by adding 10 μL of 10 M NaOH and unhydrolyzed substrate was removed by centrifuging the tubes at 4000 rpm for 5 min. Reducing sugars from the supernatants were measured using the p-hydroxy benzoic acid hydrazide (PAHBAH) method (Lever et al. 1973), with glucose as standard.

## Results

### Creation and screening of compost metagenomic libraries

Controlled composts were created using lignocellulosic materials (Whatman filter paper, steam exploded spruce and cutter chips) to enrich microbial communities producing cellulolytic and hemicellulolytic enzymes. The garden waste inoculum originated from the Ämmässuo composting plant (YTV, Finland). Composting was conducted at 37 °C and 50 °C to enrich for meso- and thermophilic organisms, respectively. The biodegradation of lignocellulose containing substrates was clearly more efficient at 50 °C. For example, after 20 weeks, 61% of cutter chips were converted to CO_2_ whereas at 37 °C the degradation was less than 20%.

Compost samples from several time-points and representing different materials used for enrichment were pooled, metagenomic DNA was extracted from the microbial communities and used to construct DNA libraries using both fosmid and plasmid vectors. The details of the constructed compost metagenomic libraries can be seen in Table 1. The primary fosmid libraries FOS-37 °C-CEL and FOS-50 °C-CEL were found to contain roughly 43.000 and 40.000 clones, respectively. Based on the number of clones containing a unique insert and the average insert size both libraries were estimated have captured about 1.2 Gb of unique sequence. The plasmid library PLA-50 °C-CEL yielded a far greater number of clones: 760,000. On average 95% of the clones contained a unique insert with an average size of 5.4 kb, bringing the total size of this library to 3.9 Gb, or about three times the size of the fosmid libraries.

**Table 1:** Details regarding the metagenomic fosmid and plasmid libraries constructed and screened for xylanase activity in this study

Library	FOS-37 °C-CEL	FOS-50 °C-CEL	PLA-50 °C-CEL
Vector	Fosmid—pCC1FOS	Fosmid—pCC1FOS	Plasmid—pCR2.1-TOPO
Number of unique clones	43,000	40,000	760,000
Average insert size	28.5 kb	30.8 kb	5.4 kb
Total library size	1226 Mb	1232 Mb	3900 Mb
No. of xylanase hits	21	18	1
Hit rate	1/58 Mb	1/68 Mb	1/3.9 Gb
Positive clone identifiers	XYL1–21	XYL22–39	XYL40

Number of clones was estimated using serial dilution and plating of the primary library. Average insert size was estimated based on restriction digests of 30–40 individual library clones

**Fig. 1:**
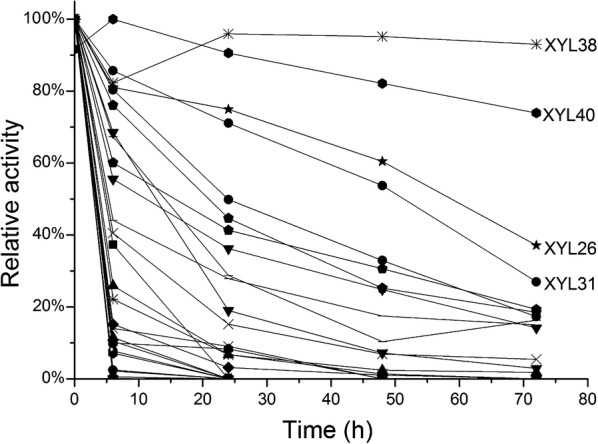
Initial thermostability assay of xylanase-positive metagenomic library clones. Whole cell lysates of the positive metagenomic clones were incubated in a water bath at 60 °C for 3 days. Samples were taken at regular intervals and residual xylanase activity quantified using the EnzCheck xylanase assay

The libraries were screened to ten times coverage of the primary library clone number on agar plates containing the chromogenic substrate AZCL-xylan. FOS-37 °C-CEL and FOS-50 °C-CEL fosmid libraries and the PLA-50 °C-CEL plasmid library yielded 21, 18 and 1 unique xylanase hit(s), respectively (Table 1). Accordingly, the hit rates for the fosmid libraries were 1/58 and 1/68 Mb, while for the plasmid library the hit rate was significantly lower at 1/3900 Mb. These numbers are in line with figures (1/14–1/1024 Mb) previously reported for endoglucanase screens (Duan and Feng 2010). However, it is not readily apparent why the hit rate for the plasmid library was so much lower than for the fosmid libraries.

### Thermostability screen of metagenomics library clones

An initial assay was devised to identify thermostable xylanases directly from cell lysates of the metagenomic library clones. We were unable to measure xylanase activity from all the lysates using birchwood xylan as substrate (Data not shown). We therefore opted for the sensitive fluorescent xylanase substrate DiFMUX_2_ (EnzCheck—Invitrogen), which allowed activity measurement in all 40 lysates. The lysates were incubated at 60 °C for a total duration of 3 days, and residual activity was quantified from samples (Fig. 1). Most lysates were found to rapidly lose their xylanase activity by 6 h of incubation, but a small number displayed considerable stability at this temperature. In particular, lysates representing two clones (XYL38 and XYL40) retained virtually all their xylanase activity for the whole duration of incubation. These clones were therefore of particular interest for further study.

### Sequencing

All unique xylanase-positive fosmid clones were pooled and sequenced using 454-pyrosequencing. This approach yielded a total of 40.6 Mb of sequence data. 9 entire fosmid inserts could be completely sequenced and assigned to specific clones using the fosmid terminal sequences generated by Sanger sequencing. Additionally, larger contigs of over 10 kb could be assigned to one of the termini of five fosmids. A further round of 454-pyrosequencing was performed on the ten most thermostable clones by LGC Genomics GmbH (Berlin). This run generated 35.1 Mb of sequence and 16 assembled contigs of over 1 kb. In total, we were able to assign over 20 kb of sequence to 20 of the fosmid clones. The single unique hit from the plasmid library (XYL40) was sequenced using Sanger sequencing.

The annotation of the sequence data set revealed 20 unique putative xylanases, of which 15 could be traced back to a particular metagenomic library clone (Table 2). Of these the clear majority (13) belonged to family GH11, while five were members of family GH10. One putative xylanase representing both families GH8 and GH30 were also found. The majority of identified genes encoded multidomain proteins, with putative carbohydrate-binding modules from families 2, 6, 22, 60 and 64 accompanying the catalytic domains. In addition, the putative xylanase in clone 21 encoded for fibronectin type III domains (FN3). FN3-domains have previously been reported in other glycosyl hydrolases, where they have been implicated in substrate binding and disruption in a fashion similar to CBMs (Kataeva et al. 2002). Finally, the GH10 xylanase in an unassigned sequence (contig 74) contained an N-terminal sequence of roughly 100 aa, with homology to cadherin-like (CADG) sequences. Bacterial CADG domains have also been shown to be active in carbohydrate binding (Fraiberg et al. 2011).

**Table 2:** The novel putative xylanase sequences discovered in the annotation of the metagenomic sequence dataset

Library	Sequence	GenBank ID	Domain organization of xylanase
FOS-37 °C-CEL	XYL1	MF171170	[GH11]-[CBM60]
	XYL3	MF171171	[GH11]-[CBM60]
	XYL7	MF171172	[GH11]-[CBM60]
	XYL11	MF171173	[GH11]-[CBM60]
	XYL12	MF171174	[GH11]-[CBM60]
	*XYL13*		
	*A*	*MF171176*	[*GH30*]
	B	MF171175	[GH11]-[CBM64]
	XYL18	MF171177	[GH10]
	XYL19	MF171178	[GH11]-[CBM60]
	*XYL21*	*MF171179*	[*GH10*]-[*FN3*]-[*FN3*]-[*CBM2*]
FOS-50 °C-CEL	XYL25	MF171180	[GH11]-[CBM2]
	*XYL32*	*MF171181*	[*GH8*]
	*XYL35*	*MF171182*	[*GH11*]
	*XYL38*	*MF171183*	[*GH10*]
PLA-50 °C-CEL	*XYL40*	*MF171184*	[*GH11*]-[*CBM60*]
Unassigned sequence	Contig. 56	MF171165	[GH11]-[CBM60]
	Contig. 74	MF171166	[CADG]-[GH10]-[CBM6]-[CBM22]-[CBM22]
	Contig. 228	MF171167	[GH10]-[CBM2] (partial)
	Contig. 1219	MF171168	[GH11]-[CBM60]
	Contig. 1238	MF171169	[GH11]

Xylanase domain structure is indicated using the abbreviations of families of glycosyl hydrolases (GH) and carbohydrate-binding modules (CBMs) in brackets. The genes selected for subcloning and further study are indicated in italics

[*CADG*] cadherin-like domain

Six enzymes were shortlisted for subcloning and purification. Three xylanases were chosen based on the initial thermostability assay: XYL40 (GH11/CBM60), XYL38 (GH10) and XYL35 (GH11). Three others were chosen due to their unique sequences. Two were the xylanases belonging to the less studied xylanase-containing families of glycosyl hydrolases, family GH8 (XYL32) and GH30 (XYL13), while one was selected due to its complex domain organization. This xylanase, XYL21 (GH10/FN3/FN3/CBM2), contained two fibronectin type III domains (FN3) linking a catalytic GH10 domain to a CBM2-type carbohydrate-binding module. An FN3-domain has previously been described in conjunction with a GH10 xylanase (Kim do et al. 2009). All the six selected xylanases were amplified from fosmid or plasmid DNA using PCR and incorporated into the pASK-IBA16 *E. coli* periplasmic secretion vector for recombinant protein production.

### Recombinant protein production

**Fig. 2:**
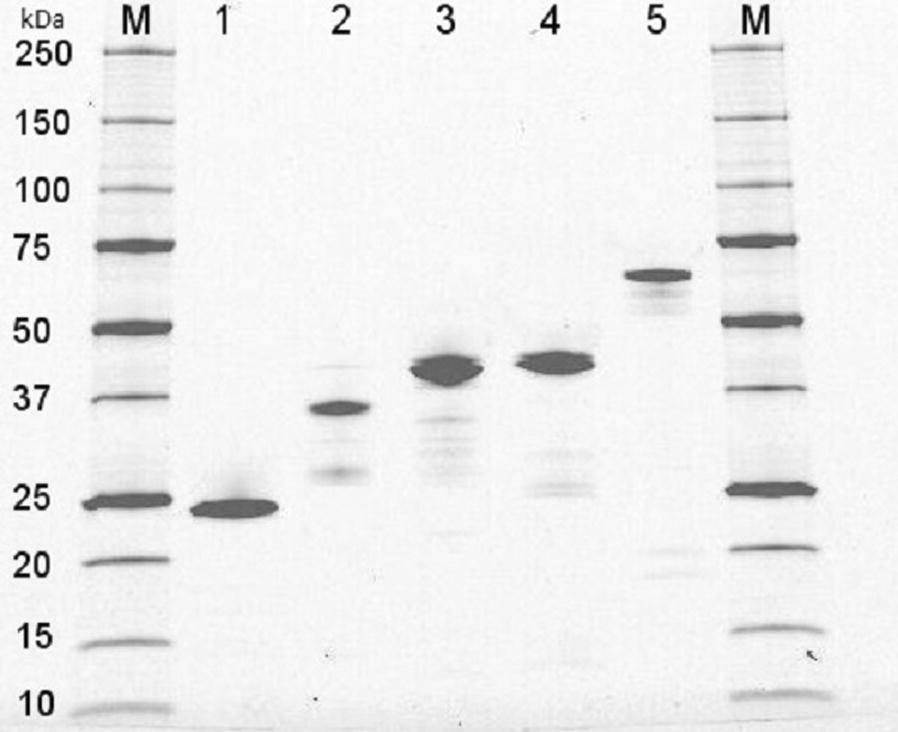
SDS-PAGE analysis of the five purified recombinant metagenomic xylanases. Two micrograms of each purified enzyme was loaded on a 4–20% Criterion™ TGX Stain-Free™ Precast gradient gel (Bio-Rad) and visualized on a Bio-Rad Criterion Strain-free imaging system. Lanes: M—Prestained Precision Plus protein standard (Bio-Rad), 1—XYL35 (GH11), 2—XYL40 (GH11-CBM60), 3—XYL38 (GH10), 4—XYL32 (GH8), 5—XYL21 (GH10 + FN3 + FN3 + CBM2)

The recombinant xylanases were produced in *E. coli* shake flask cultures and the protein recovered by periplasmic extraction and purified using StrepTag affinity chromatography. The approach yielded sufficient amounts (> 1 mg) of recombinant protein for preliminary characterization of five of the selected xylanases (Fig. 2). The five recombinant enzymes were found to be active on both the small-molecule EnzCheck substrate and birchwood xylan (Roth). Only in the case of the family GH30 enzyme (XYL13) the protein yields and quality were not sufficient to allow the study of enzyme properties.

### Preliminary characterization of novel xylanases

**Fig. 3:**
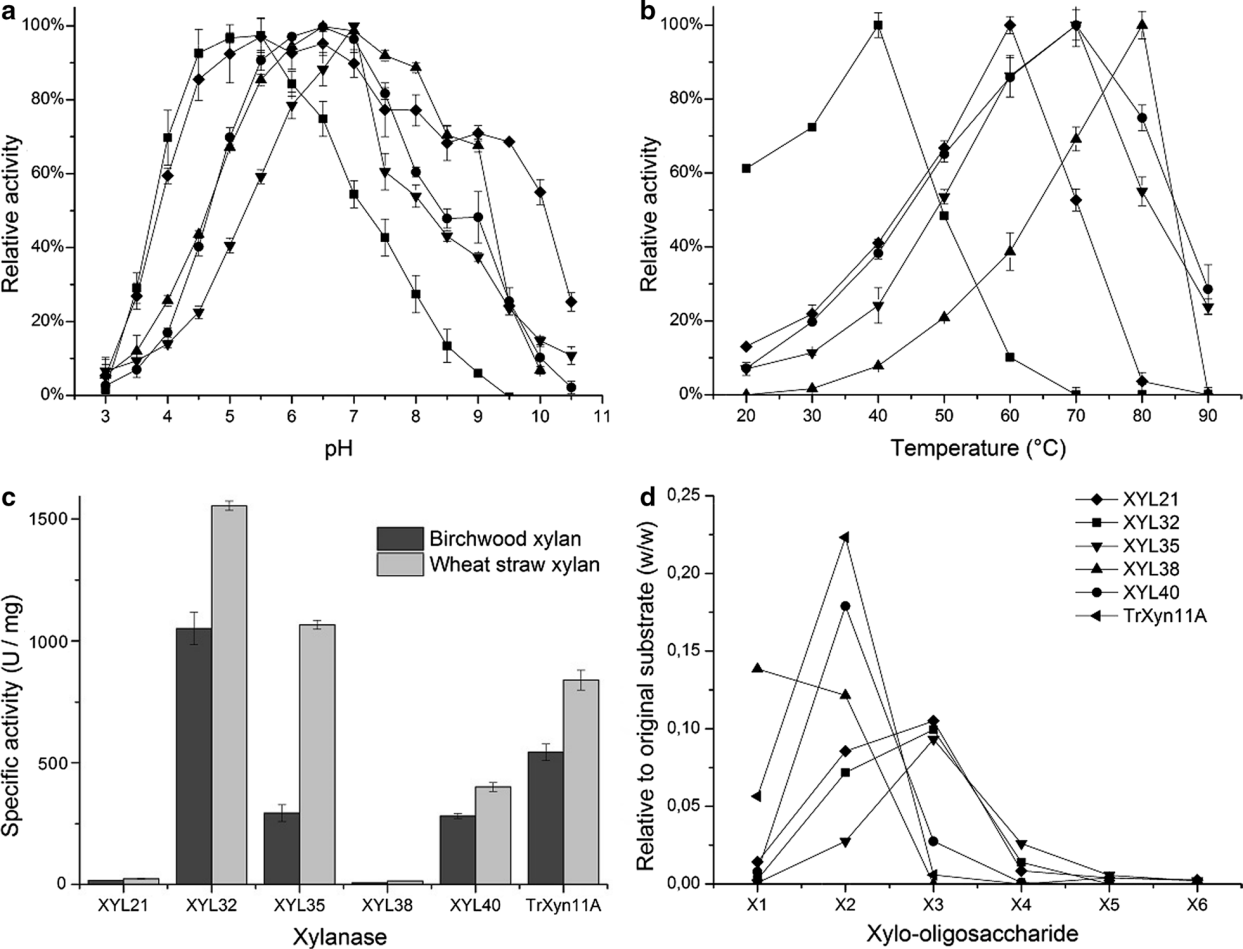
Preliminary characterization of the purified recombinant xylanases. **a** The relative activity of the purified recombinant xylanases on birchwood xylan as a function of pH. **b** Temperature optima were measured by conducting the standard xylanase assay at different temperatures over a range of 20 °C to 90 °C. **c** Specific activity of the purified xylanases on birchwood glucuronoxylan (GX) and wheat arabinoxylan (AX). **d** Xylo-oligosaccharides (X_1_–X_6_) released by recombinant xylanases from birchwood glucuronoxylan as measured by HPLC. Results plotted as relative to original substrate mass (w/w)

The purified recombinant xylanases were characterized in terms of temperature and pH optima, thermostability, substrate specificity and hydrolysis profile. The results are summarized in Fig. 3 and in Table 3.

**Table 3:** Characteristics of the purified recombinant xylanases

Xylanase	XYL21	XYL32	XYL35	XYL38	XYL40
Domain organization	[GH10]-[FN3]-[FN3]-[CBM2]	[GH8]	[GH11]	[GH10]	[GH11]-[CBM60]
Size (aa)^a^	602	385	196	370	325
Mw (kDa)^a^	63.2	44.3	24	41.9	34.9
pI^a^	7.5	5.4	9.8	6.9	8.1
pH optimum	5.5	5.5	7.0	6.5	6.5
Temp. optimum	60	40	70	80	70
XOS produced from GX	X_2_/X_3_	X_2_/X_3_	X_2_–X_4_	X_1_/X_2_	X_2_
GX (U/mg)	17.2	1048	293	7.1	281
AX (U/mg)	23.8	1551	1062	13.8	399
DiFMUX_2_ (U/mg)	0.110	0.0072	10.0	0.106	1.94
Symbol used in legends	♦	■	▼	▲	●

^a^Figures presented for molecular weight and pI were calculated based on the amino acid sequences of the native secreted proteins excluding the purification tag

*GX* birchwood glucuronoxylan, *AX* wheat straw arabinoxylan

The pH profiles of the purified xylanases were assayed over a pH range 3.0–10.5 (Fig. 3a). None of the novel enzymes was active at the acidic pH of 3, but the xylanases XYL32 and XYL21 show considerable activity at pH 4 with 70% and 60% activity, respectively. Both of these enzymes also have relatively low pH optima. The pH optimum of XYL32 was 5.0–5.5, while XYL21 had a very broad pH profile in general, with maximal activity occurring over a range of pH 5–7 and still retaining over 50% activity at pH 10. The most thermostable enzymes based on the initial characterization, XYL38 and XYL40, had very similar pH profiles with peak activity occurring at pH 6.5. XYL38 fared slightly better at a more alkaline pH, retaining over 70% activity at pH 9. XYL35 had the narrowest pH profile with a pH-optimum of 7.0.

The optimal temperature for all the purified xylanases was measured by performing reactions at different temperatures over a range of 20 °C to 90 °C (Fig. 3b). The highest value found was 80 °C for XYL38, followed by 70 °C for both XYL40 and XYL35. Interestingly, XYL32 displayed high activity at 20 °C and could therefore be suitable for application where cold-active enzymes are required.

The activity of the enzymes was additionally quantified on wheat arabinoxylan and carboxymethyl cellulose (CMC). CMC was included as a control for possible endoglucanase activity. *T. reesei* Xyn11A was included as a reference enzyme in this assay. Higher activities were measured on wheat straw arabinoxylan than on birchwood glucuronoxylan for all enzymes. This substrate preference was more striking in the case of XYL35, which had over threefold higher activity on wheat straw xylan. XYL21 was the only enzyme for which CMCase activity could be detected under these assay conditions. The specific activity on CMC (1.8 U/mg) was over tenfold lower than that observed for arabinoxylan.

Figure 3c also allows comparison of the specific activities of the different enzymes. Interestingly both enzymes belonging to family GH10, XYL21 and XYL38, had significantly lower specific activities than the other enzymes, 17.4 and 7.2 U/mg, respectively. All enzymes belonging to family GH11, including the reference enzyme TrXyn11A, had specific activities on birchwood xylan in the 240–500 U/mg range, while the specific activity of the GH8 xylanase XYL32 was nearly twice that of TrXyn11A at 1050 U/mg. We also quantified the specific activities of the purified xylanases on the DiFMUX_2_ model substrate (Table 3). A difference in specific activities of over 10^3^-fold was observed with this substrate, with the GH8 xylanase XYL32 having clearly the lowest level of activity on this substrate.

The hydrolysis products generated by the metagenomic xylanases from birchwood xylan were analyzed using high-performance liquid chromatography (HPLC). The GH10 xylanase XYL38 was found to degrade the xylan to the furthest extent, with xylose (X_1_) being the most abundant reaction product in the hydrolysate (Fig. 3d). In our assay, the GH11 enzymes XYL40 and TrXyn11A had very similar hydrolysis profiles, with xylobiose (X_2_) being the dominant reaction product. The hydrolysis products produced by the three remaining enzymes (XYL21, XYL32 and XYL35) contained primarily xylobiose and -triose.

**Fig. 4:**
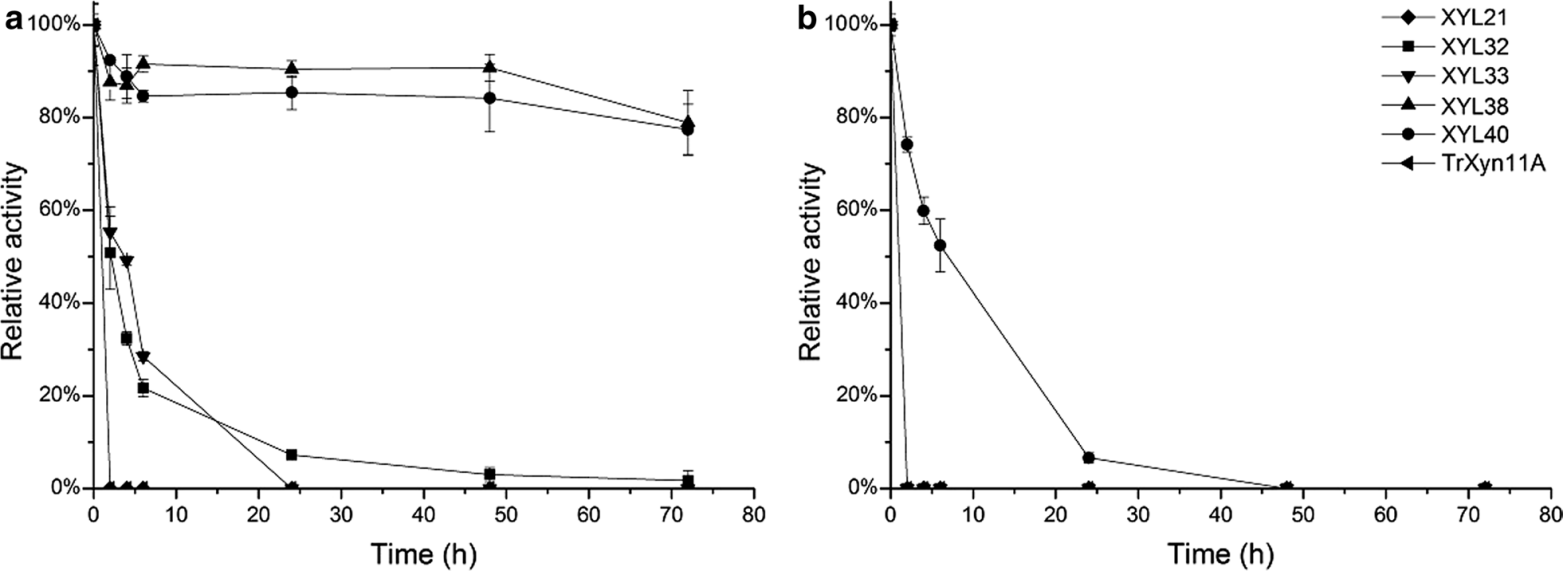
Stability of the purified metagenomic xylanases at 60 °C (**a**) and 70 °C (**b**). The purified enzymes were incubated in water baths at 60 °C (**a**) and 70 °C (**b**) for a total duration of 3 days and residual xylanase activity was quantified using the standard assay

The thermostability of the purified recombinant enzymes was quantified at 60 °C and 70 °C (Fig. 4). The reference TrXyn11A and the multidomain XYL21 were found to lose all activity at 60 °C by the first time-point (2 h). XYL32 and XYL35 were of intermediate stability, gradually losing most of their activity during the first 6 h of incubation. XYL38 and XYL40 were found to be equally stable in purified recombinant form as in the initial assay conducted on the *E. coli* whole cell lysates (Fig. 1). Both enzymes remained virtually stable at 60 °C, while XYL40 was found to remain remarkably stable even at 70 °C, with a half-life of roughly 6 h.

### Hydrolysis of pre-treated wheat straw

**Fig. 5:**
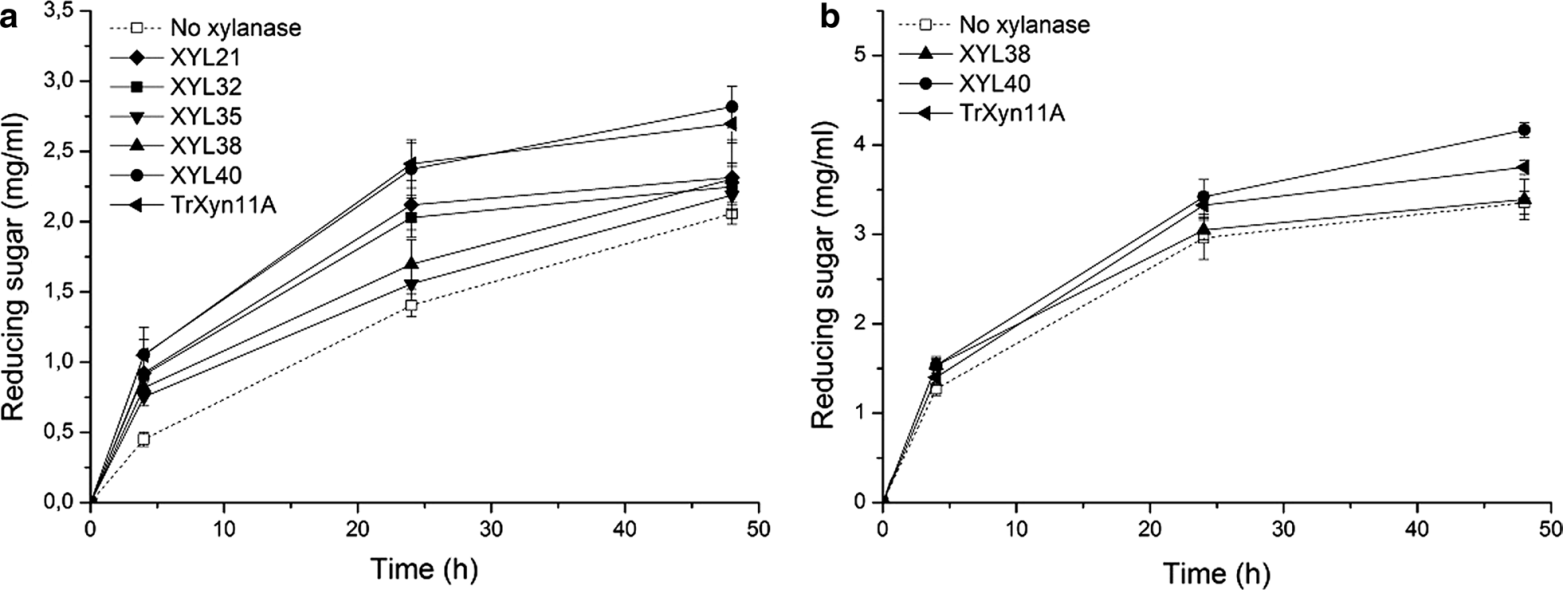
Hydrolysis of hydrothermally pre-treated wheat straw using novel xylanases as components of multienzyme mixtures. **a** Hydrolysis at 45 °C using mesophilic enzymes: *T. reesei* cellulases (TrCel7A, TrCel5A) and *A. niger* glucosidase (AnCel3A), supplemented with xylanase according to figure inset. **b** Hydrolysis at 55 °C using thermostable enzymes: CBHI from *Acremonium thermophilum* (AtCel7A), CBHII from *Clostridium thermocellum* (CtCel6A) in addition to EGII and β-glucosidase from *Thermoascus aurantiacus* (TaCel5A and TaCel3A). Xylanase was included according to figure inset

The performance of the purified recombinant xylanases in the degradation of hydrothermally pretreated wheat straw was evaluated using model enzyme cocktails (Fig. 5). Hydrolysis was performed at 45 °C and 55 °C, combining the novel xylanases with the core cellulases of *T. reesei* and thermostable enzymes provided by Roal Oy, respectively.

All xylanases improved the hydrolysis of pretreated wheat straw at 45 °C compared to the reference cellulases alone (dashed line in Fig. 5a). The increase was most notable at the initial 4 h time point. After this time point the xylanases could be divided into three groups based on performance: The best hydrolysis yields were achieved when the cellulases were supplemented with TrXyn11A or the most thermostable xylanase XYL40. The lowest yields were achieved using XYL35 or XYL38, with only a minor improvement over the core cellulase mixture. The performance of XYL32 and XYL21 could be described as intermediate. It is interesting to note that the enzymes that performed the best in this assay, TrXyn11A and XYL40, share a very similar reaction rate and hydrolysis profile (Fig. 3d).

The effect of xylanase addition was also clearly observable using the mixture of thermostable enzymes at 55 °C (Fig. 5b). XYL40 and the TrXyn11A provided similar yields up to 24 h, after which XYL40 performed better, most probably due to inactivation of TrXyn11A. XYL38 surprisingly failed to increase yields at all after 4 h. The result suggests that XYL40 could be a useful thermostable xylanase for use in lignocellulose degradation, while for XYL38 its poor specific activity hinders its use.

## Discussion

In the present study, we set out to identify novel xylanases desirable characteristics, with particular emphasis on thermostable enzymes that are considered advantageous for many applications (Kumar et al. 2018). Metagenomics offers an attractive toolset for the discovery of enzymes with novel backbones and/or improved characteristics. Activity-based plate screening of metagenomic libraries is an efficient method for identifying desired enzymes that are expressible in the cloning host, but screening large DNA-libraries can quickly become very labor-intensive. We aimed to lower the number of clones needed to be screened (increased hit-rate) by first enriching desired microbial species using target carbon sources. Enriching has previously been applied using lignocellulosic materials such as rice straw (Reddy et al. 2013), switchgrass (Allgaier et al. 2010), Napier grass (Kanokratana et al. 2018) and crystalline cellulose (Mori et al. 2014).

The number of clones to be screened can also be lowered by increasing the amount of sequence per clone, e.g. by using large-insert fosmid vectors (Colombo et al. 2016; Lewin et al. 2017). However, in large-insert vectors the enzymes are expressed from their native promoters, which can result in low expression in the cloning host (*E. coli*). In our study, reducing sugar-based xylanase activity assays could not detect activity from several of our fosmid clones. A fluorescence-based microplate assay proved more sensitive and allowed early insight to the thermostability of the uncovered xylanases. Using this data we shortlisted two GH11 xylanases (XYL40 and XYL35) and one GH10 xylanase (XYL38) for further study. Two other xylanases (XYL21 and XYL32) were also subcloned and studied in more detail due to their rather unique primary sequences.

The recombinant XYL38 (GH10) and XYL40 (GH11) proved to be very stable at 60 °C, and the latter even retained activity for long periods at 70 °C. Although several more thermostable enzymes have been reported (Kumar et al. 2018), few have been tested in lignocellulose hydrolysis as components of cellulolytic enzyme cocktails. XYL40 was found to perform better that Xyn11A from *T. reesei* in hydrolysis experiments carried out on an authentic lignocellulosic substrate at 55 °C. This enzyme therefore shows potential toward lignocellulose hydrolysis as a component of a cocktail of thermostable enzymes.

The other thermostable enzyme XYL38 performed rather poorly in the wheat straw hydrolysis experiments. This enzyme had a very low specific activity toward birch wood and wheat straw xylans, from which it released xylose in addition to xylobiose, and showed activity on carboxymethylcellulose. GH10 enzymes generally have broad substrate specificities, and activity on cellulose has been reported (Chu et al. 2017; Wang et al. 2019). The release of xylose as the main product suggests XYL38 might display exo-activity (Juturu and Wu 2014). The data suggests that XYL38 is a rather unusual xylanase and considerably different from its closest characterized homologue Xyn10A from *Acidothermus cellulolyticus* 11B (50.3% identity). This enzyme was inactive on CMC and did not produce detectable amounts of xylose from birchwood xylan (Barabote et al. 2010). The other recombinantly produced GH10 and GH11 xylanases, XYL21 (GH10-FN3-FN3-CBM2) and XYL35 (GH11) did not display any particularly attractive properties considering potential industrial applications.

We also expressed a novel xylanase belonging to the less-studied family GH8 (XYL32). This enzyme had the highest specific activity among the tested xylanases on both types of polymeric xylan. It also had the lowest temperature optimum (40 °C) among all the xylanases, and retained over 60% of this activity at 20 °C. Its high activity at low temperature and acidic pH optimum (5.5) might make it suitable for e.g. certain food applications (Dornez et al. 2011). The enzyme also showed low but detectible activity on the fluorescent EnzCheck substrate. Another GH8 xylanase was previously found to be completely inactive on the substrate (Ge et al. 2007). The hydrolysis profile of XYL32 (Fig. 3d) suggests that it is clearly an endo-acting xylanase. It shares 42% and 43% identity with two characterized GH8 endo-xylanases, Xyn8A from *Bacteroides intestinalis* (Hong et al. 2014) and TtGH8 from *Teredinibacter turnerae* (Fowler et al. 2018), both of which also released xylotriose as their main hydrolysis product from xylan.

In addition to xylanases, our dataset comprised a significant amount of sequence with numerous putative ORFs encoding additional carbohydrate active enzymes (CAZymes), several of which are related to the depolymerization of xylan. For example, a genomic insert (GenBank KX236224.1) containing the most thermostable xylanase XYL40, also encoded a putative GH115 α-d-glucuronidase, GH51 α-l-arabinofuranosidase and a GH9 endoglucanase. This demonstrates one of the strengths of combining functional screening of large-insert vectors with high throughput sequencing: Using this approach it is possible to uncover whole operons of genes with interrelated functions, and discover novel enzymes for which plate-screening substrates might not be available.

In summary, we report here the use of environmental enrichment using controlled composting of target materials, followed by the construction and screening of metagenomic DNA libraries for novel xylanases. Five novel xylanases were expressed, purified and preliminarily characterized. All enzymes were found to be active not only on screening and small-molecule substrates, but also on the technical lignocellulosic feedstock wheat straw, with one enzyme (XYL40) displaying superior performance in wheat straw hydrolysis compared to TrXyn11A.

## Data Availability

The sequence data described in this article is deposited and accessible at GenBank.
